# The American Journal of Science and Arts

**Published:** 1850-03

**Authors:** 


### REVIEWS.

---

Art. V.—*The American Journal of Science and Arts.* By Professor B. Silliman, L. L. D., M. D., &c., &c. Prof. B. Sillimam, Jr., A. M., M. D., &c., and James D. Dana, A. M. New Haven, Conn. No. 25, for January, 1850.

More than a generation of men has passed away since the veteran and pioneer of American science whose name still stands on the covers of this Journal, issued its first number.

In that time it has gone uninterruptedly forward to the completion of its first series of fifty volumes in 1845; and the second series has now reached its ninth volume. In 1817, Dr. Archibald Bruce, of New York, issued the first and only volume of the "American Mineralogical Journal," but his premature death arrested the continuation of the work so well begun. With this solitary exception, the "American Journal of Science," was the first Scientific Journal in this country, exclusively devoted to the pure sciences. And it has remained longer under one editor, issued on its original plan, in one series, than any other Journal, scientific or literary, issued in the English language.

It has ever been characterized, from the commencement, by its just, manly, and independent course, attributes which might have been anticipated from the well known urbanity and uprightness of its projector and senior editor.

Prefacing the fiftieth volume of the first series, is a lucid and even eloquent history of the origin and progress of this great American enterprize, which must be read, by every American, with emotions of just pleasure and admiration.

Our readers will need no apology from us, if we use, in this notice, the facts there given, either in the words of the author, or our own rescript.

It is worthy of remark, that the projector, in the very outset, showed the comprehensiveness and enlargement of his mind, and the just sense he felt of the nature of his task, when he announced, in his first number, the "Plan of the Work." "This Journal" he says, "is intended to embrace the circle of the physical sciences, with their applications to the arts, and to every useful purpose." He thus broadly threw open the door for collaborators in all the numerous departments in the varied fields of physical investigation, and the result has shown the wisdom of his course, for up to the close of the first series, not less than six hundred names were reckoned among the original contributors, who had made this Journal the vehicle of their communications. We are assured, in this preface, that of this large army of scientific men, naturalists, and philosophers, over five hundred were, in 1847, still upon the stage of action, and, indeed, that only some fifty were then certainly reckoned among the dead. "Among them are not a few of the veterans with whom we began our career, and several of these are still active contributors. Shall we then conclude that the peaceful pursuits of knowledge are favorable to long life? This, we think, is, cæteris paribus, certainly true: but in the present instance, another reason can be assigned for our large amount of survivorship. As the Journal has advanced, and death has removed its scientific contributors, younger men, and men still younger, have recruited the ranks, and volunteers have enlisted in numbers constantly increasing, so that the flower of the host are now in the morning and meridian of life.

"We have been constantly advancing, like a traveler from the equinoctial towards the colder zones—as we have increased our latitude, stars have set and new stars have risen, while a few planetary orbs, visible in every zone, have continued to cheer us on our course.

"The number of articles, almost exclusively original, contained in the Journal, is about 1800, and the index shows how many have been contributed by each individual. * * * * * Of party, either in politics or religion, there is no trace in our work; of personalities there are none, except those that relate to priority of claim, or other rights of individuals. Of these vindications the number is not great, and we could heartily have wished that there had been no occasion for any."

For twenty years, from the inception of this Journal, the editor labored alone, although overtures for editorial coöperation, had been made to him, by gentlemen commanding his confidence and esteem, and who would personally have been very acceptable. It was, however, his opinion that the unity of purpose and action so essential to the success of such a work, were best secured by *individuality*,—but he has made every effort, and not without success, to conciliate the good will and secure the assistance of gentlemen eminent in particular departments of knowledge. In 1838, however, the second of the present editors appeared, for the first time, upon the title page of the July number for that year.

Speaking of this second name, the preface says: "The individual, to whom it belongs, having been, for several years, more or less concerned in the management of the Journal, and from his education, position, pursuits and taste, as well as from affinity, being almost identified with the editor, he seemed quite a natural ally, and his adoption into the editorship was scarcely a violation of individual unity."

The patronage of this Journal, up to the close of the first series, had never reached one thousand paying subscribers, and has rarely exceeded seven or eight hundred. Since the institution of the second series, and alteration of the times of issue, (now six times yearly, in place of the quarterly mode) it has increased somewhat in its circulation, but we understand that the increased cost of the new issue leaves the pecuniary result scarcely, if at all, better than of old.

It has been far from paying a reasonable editorial compensation; it has often paid nothing, and never did much more than pay its bills.

The large number of engravings, and the extra labor in printers' composition, cause it to be an expensive work, while its patronage is limited. In the first series there are nearly three hundred copperplates, and over ten times that number of wood cuts.

But the increasing love of science in this country, the systematic efforts made for its advancement, and the general prosperity, will not long permit this invaluable Journal to feel itself hampered by the want of means. The first yearnings of a love for science and philosophy should manifest itself by a subscription to Silliman's Journal, and those who have reached the greatest heights of science should feel themselves as much bound to subscribe for the Journal of Science, as to do anything else of a scientific character. There is no one way in which science may be as much promoted as in enlarging the usefulness of Silliman's Journal. To that great object every devotee of science should devote his energies.

We have already referred to two of the names of the editorial corps of the Journal of Science, but we should do great injustice to our feelings of grateful admiration, if we were to omit all reference to the third name—that of James D. Dana. He is, in many respects, the most profound thinker and the most admirable writer we have ever followed in scientific investigations. His inductive power is so remarkable, that he is almost as bold in his inductions, as Leibnitz was in his speculations. As a geological essayist, we think he has no equal; at any rate, we have seen nothing on geology that is to be compared with the remarkable series of papers he has published in the Journal of Science.

The high character of Mr. Dana pointed him out to the government as the Geologist of the United States Exploring Expedition, and never was any public trust more completely fulfilled. There is not a people in the world, —not even France, with all her scientific glories challenging the admiration of the world—nor England, with all the fullness of her scientific fame—that would not feel proud of the works of Dana, connected with the Exploring Expedition.

The pages of the Journal of Science often feel the impress of Mr. Dana's genius, and of his clear and cogent style. His style, as a writer, is peculiar. Each governing word in his argument, his facts, and his inductions is as clear as crystal.

We urge upon our readers to subscribe for Silliman's Journal. Under the editorial supervision of the elder Silliman, it attained its position as one of the foremost scientific journals of the world, and under the management of its present editorial corps, we think it has no ri val anywhere. Practitioners of medicine can do themselves no harm by a diligent study of the sciences elaborated in Silliman's Journal, but, on the contrary, they may greatly enlarge their usefulness in their profession.

T. R. Nelson, bookseller, is the agent for this work in Louisville, but the mail is the best communication for country subscribers, direct from the home office.

